# First-trimester prediction of early-onset preeclampsia using PAPP-A and mean arterial pressure

**DOI:** 10.17305/bb.2025.12814

**Published:** 2025-07-23

**Authors:** Fatma Beyazıt, Eren Pek, Murat Daş, Mehmet Nuri Duran, Dilek Ülker Çakır, Başak Nil Şen, Hasan Ali Kiraz, Deniz Koçyiğit Yılmaz, Ece Ünal Çetin

**Affiliations:** 1 Çanakkale Onsekiz Mart University, Medical School, Department of Obstetrics and Gynecology, Çanakkale, Türkiye; 2 Çanakkale Onsekiz Mart University, Medical School, Department of Emergency Medicine, Çanakkale, Türkiye; 3 Ezine State Hospital, Obstetrics and Gynecology Clinic, Çanakkale, Türkiye; 4 Çanakkale Onsekiz Mart University, Medical School, Department of Medical Biochemistry, Çanakkale, Türkiye; 5 Çanakkale Onsekiz Mart University, Medical School, Department of Anesthesiology, Çanakkale, Türkiye; 6 Ipsala State Hospital, Obstetrics and Gynecology Clinic, Edirne, Türkiye; 7 Çanakkale Onsekiz Mart University, Medical School, Department of Internal Medicine, Çanakkale, Türkiye

**Keywords:** Prediction, preeclampsia, PE, pregnancy, pregnancy-associated plasma protein-A, PAPP-A, hypertension, HT

## Abstract

Predicting early-onset preeclampsia (EOP) during the initial stages of pregnancy is essential for effective clinical management and enhancing maternal-fetal outcomes. Current methodologies, which include clinical and demographic risk factors, biophysical parameters, and serum biomarkers, exhibit limited efficacy in predicting EOP. This study aimed to evaluate whether the incorporation of pregnancy-associated plasma protein-A (PAPP-A) and mean arterial pressure (MAP) significantly enhances EOP detection. We conducted a retrospective case-control study involving 518 gravidas, of whom 202 developed EOP and 316 experienced normal pregnancies. Logistic regression models were employed to assess EOP predictions, and the predictive accuracy of these statistical models was evaluated using receiver-operating characteristic curve analysis. Our findings indicate that lower PAPP-A levels, higher MAP, and increased body mass index (BMI) are associated with EOP. Notably, in pregnant women between 11+0 and 13+6 weeks of gestation, a 1-point decrease in PAPP-A corresponds to an 84% increase in the likelihood of developing EOP. The predictive performance of PAPP-A improves significantly when combined with other factors such as BMI, MAP, and a history of diabetes mellitus (DM). The risk of EOP is substantially heightened (20.410 times, 95% CI: 11.104–37.515) in patients exhibiting low PAPP-A levels (<0.88) and high BMI (≥35 kg/m^2^). Additionally, low PAPP-A combined with elevated MAP levels significantly increases EOP risk (adjusted odds ratio [OR]: 114.83). However, after adjustment, the association between low PAPP-A and a history of DM was not statistically significant (adjusted OR: 2.30, *P* ═ 0.202). In conclusion, employing a combination of multiple variables for predicting EOP yields a significant improvement over traditional methods that rely solely on individual factors.

## Introduction

Preeclampsia (PE) is a multisystemic disorder that develops during pregnancy, typically after the 20th week of gestation. It is characterized by new-onset hypertension (HT) accompanied by proteinuria or by HT with progressive end-organ dysfunction, even in the absence of proteinuria [1]. PE can be categorized into two types based on the timing of onset: early-onset PE (EOP), requiring delivery prior to 34 weeks of gestation, and late-onset PE (LOP), occurring with delivery at or beyond 34 weeks of gestation [2]. This multisystem disorder poses significant risks to both the mother and the developing fetus, including complications like premature birth, low birth weight, and, in severe scenarios, critical maternal conditions such as seizures or organ failure [3]. Therefore, early identification of PE is critical, as it enables timely decision-making for intervention and management, reducing the likelihood of adverse outcomes. Routine prenatal visits, monitoring blood pressure, and urine testing are key components of early detection, allowing clinicians to implement appropriate measures to protect maternal and fetal health. Regrettably, numerous challenges and limitations persist in the early prediction and management of PE. Thus, it is crucial to develop validated clinical, laboratory, and radiological risk prediction tools to improve the detection of PE, particularly during the first prenatal visit in the first trimester.

Over the past decade, there has been a growing focus on predictive clinical and biochemical markers for PE. An effective predictive test could streamline prompt diagnosis, enable targeted surveillance, and facilitate proper management with timely delivery. In this context, the single or combined use of several prediction tools, including pregnancy-associated plasma protein-A (PAPP-A), maternal mean arterial pressure (MAP), serum levels of placental growth factor (PlGF), uterine artery pulsatility index, and maternal demographic factors—including age, history of PE, diabetes mellitus (DM), and body mass index (BMI)—has been extensively investigated [4, 5]. Among these, PAPP-A and MAP have been most extensively studied [6, 7]. PAPP-A is a glycoprotein secreted by the syncytiotrophoblast and decidua. Low levels of PAPP-A in chromosomally normal pregnancies are linked to an elevated risk of subsequently developing PE. Given the presumed significant role of insulin-like growth factor in trophoblast invasion, the relationship between low serum PAPP-A and an increased incidence of PE is not unexpected [8]. Furthermore, studies have shown that combining MAP and PAPP-A significantly improves the prediction of PE [5, 9]. However, whether adding these parameters to maternal characteristics improves prediction effectiveness remains controversial.

To address this knowledge gap, we aimed to create and validate a multivariable risk prediction tool for EOP by integrating maternal MAP, PAPP-A, and several well-known maternal clinical and demographic risk factors measured at 11–14 weeks of pregnancy. We hypothesized that early pregnancy MAP and PAPP-A, combined with maternal history, biometric variables, and biophysical factors, would provide a more accurate prediction of EOP compared to traditional approaches.

## Materials and methods

### Study design and patient eligibility

This retrospective case-control study was carried out at Çanakkale Onsekiz Mart University (COMU) Training and Research Hospital. The study enrolled women who underwent prenatal screening at the COMU Prenatal Screening Center during 11–13+6 weeks of gestation and gave birth at the same institution between January 2016 and June 2023.

In line with the retrospective case-control design, all available cases of EOP during the study period (*n* ═ 202) were included. A control group of 316 women with uneventful pregnancies was randomly selected from the same hospital population and time frame. The final analysis comprised a total of 518 participants, including 202 pregnant women with EOP and 316 healthy controls, all of whom met the specified inclusion criteria. The following parameters were recorded from medical files for each participant: personal and family history (age in years, height, and body weight; parity/gravidity; history of PE, DM, and HT), biophysical factors (BMI; systolic blood pressure [SBP] and diastolic blood pressure [DBP]), and serum PAPP-A levels. The control group comprised 316 healthy pregnant women who experienced a normal delivery and gave birth to a single live infant within the Obstetrics and Gynecology Department of COMU Hospital during the same timeframe. Ethical approval for the study was granted by the COMU local ethics committee (No: 2023-YÖNP-173:2023/14-16).

### Biophysical measurements

BMI was calculated by dividing the body weight in kilograms by the square of the height in meters, rounded to one decimal place. MAP was measured using a brachial sphygmomanometer (Erka GmbH, Bad Toelz, Germany) calibrated every 6 months in accordance with institutional standards. Maternal MAP was determined using the formula: [Systolic blood pressure (SBP)+2×Diastolic blood pressure (DBP)]/3. Blood pressure was assessed on the right arm, with the participant seated, after a 5-min rest period. Measurements were taken at least two times.

### Measurement of PAPP-A and other laboratory parameters

Maternal serum samples were collected during routine first-trimester aneuploidy screening, and the concentrations of PAPP-A and other laboratory parameters were quantified. PAPP-A levels were measured using a time-resolved fluoroimmunoassay on the automated IMMULITE^®^ platform (Siemens Healthcare Diagnostics, Gwynedd, UK), in accordance with the manufacturer’s instructions. Internal quality-control procedures were implemented throughout the assay process and met the laboratory’s established standards. The resulting PAPP-A concentrations were converted into multiples of the median (MoM) using the Prisca software (Typolog, Germany), after adjustment for gestational age, fetal crown–rump length, maternal weight, parity, smoking status, method of conception, and other relevant maternal characteristics. Routine hematological and biochemical analyses were performed for all study participants. CBC measurements were conducted in the hematology laboratory using an automated analyzer from Beckman Coulter (High Wycombe, United Kingdom).

### Diagnostic considerations

The diagnosis of PE was established if pregnant women displayed an SBP of ≥140 mmHg and/or DBP of ≥90 mmHg on at least two occasions after 20 weeks of gestation, in individuals who were normotensive before. The presence of elevated blood pressure had to be accompanied by at least one of the following new-onset conditions: 1) urinary protein excretion ≥300 mg/24 h, or random urinary protein ≥ (++); 2) evidence of maternal organ dysfunction involving the heart, lungs, liver, and kidneys; 3) uteroplacental dysfunction, as per FIGO guidelines, encompassing fetal growth restriction, abnormal umbilical artery Doppler waveform analysis, or stillbirth [10]. Pregnant women who met this criterion for PE and had deliveries occurring before 34^+0^ weeks of gestation were further subclassified into EOP.

The criteria for exclusion were outlined as follows: multifetal gestation; concurrent medical conditions including pre-gestational DM (type 1 or type 2), chronic HT, cardiac ailments, kidney disease, hyperthyroidism, autoimmune disorders, and hematological conditions; infants conceived *in vitro*; congenital anomalies in the fetus; incomplete data; and those on antihypertensive medication. However, patients with a history of gestational DM (GDM) were not excluded, as GDM is typically diagnosed later in pregnancy and may represent a relevant risk factor for EOP.

### Ethical statement

This study was approved by the Ethics Committee of Çanakkale Onsekiz Mart University Faculty of Medicine (Approval no: 2023-YÖNP-173:2023/14-16). All procedures involving human participants were conducted in accordance with the ethical standards of the institutional research committee and with the 1964 Helsinki Declaration and its later amendments or comparable ethical standards. Written informed consent was obtained from all participants.

### Statistical analysis

In this study, our descriptive statistical methodology was as follows: for variables that did not adhere to a normal distribution, we computed the median and interquartile range (IQR). Nominal variables were expressed as counts and percentages. The Shapiro–Wilk test was used to evaluate the normality of data distribution. Differences in median values between groups were assessed using the Mann–Whitney *U* test. For categorical variables, Pearson’s chi-square test or Fisher’s exact test was applied, depending on the specific characteristics of the data.

To evaluate risk factors for EOP, we computed odds ratios (ORs) with 95% confidence intervals using both univariable and multivariable logistic regression models. Variables with a *P* value <0.10 in the univariable analysis were selected for entry into the multivariable logistic regression model, using a stepwise forward selection strategy based on the likelihood ratio.

We computed ORs for EOP development, along with 95% confidence intervals, for various clinical parameters using both univariable and multivariable logistic regression models. These models played a crucial role in predicting specific outcomes such as EOP.

The goodness-of-fit of the final models was evaluated using the Hosmer-Lemeshow test. A lack-of-fit *P* value greater than 0.05 (not significant) indicated that the model had a good fit. Additionally, covariate-adjusted receiver operating characteristic (ROC) curve analysis was performed using multivariable logistic regression to assess diagnostic accuracy and calculate the areas under the receiver operating curves (AUROC). Pairwise comparisons of the AUROC values for each prediction model were conducted using the DeLong test. Multicollinearity among independent variables in the multivariable model was evaluated using variance inflation factor (VIF) statistics. All included variables demonstrated acceptable multicollinearity, with VIF values remaining below the threshold of 2.5. The optimal cut-off thresholds for PAPP-A, MAP, and BMI were empirically derived from ROC curve analyses using the Youden Index criterion to identify the values maximizing combined sensitivity and specificity in distinguishing cases from controls. Statistical analyses were carried out using SPSS 20.0 for Windows (IBM Corp., Armonk, NY, USA). A *P* value < 0.05 was considered statistically significant.

## Results

Overall, 202 pregnant women aged over 18 years who developed EOP and 316 healthy pregnant women were enrolled. The median age of the patients and controls was 30.0 years (range: 26.0–35.0 years) and 31.0 years (range: 27.0–34.0 years), respectively (*P* ═ 0.245). Although the median BMI did not differ significantly between the EOP group and controls (*P* ═ 0.057), higher BMI levels, particularly BMI ≥ 35 kg/m^2^, were significantly more common in the EOP group (*P* < 0.001). Statistical analysis indicated that higher MAP levels were also significantly associated with an increased risk of EOP (*P* < 0.001). Table 1 provides a detailed comparison of selected demographic and clinical characteristics of the patients and controls. HDL and triglyceride levels were found to be lower in the EOP group. Furthermore, PAPP-A levels were significantly lower (*P* < 0.001) in patients with EOP. Other routine hematological and biochemical data for study participants are presented in Table 2.

Both univariable and multivariable logistic regression analyses were performed to assess the influence of specific clinical and laboratory parameters in predicting the development of EOP, as outlined in Table 3. Univariable analysis revealed that a BMI ≥ 35 increased the risk of EOP by 9.506 times (*P* < 0.001). Moreover, a history of PE and being primigravida increased the risk of EOP by 10.022 and 3.867 times, respectively. Among pregnant women at 11–13+6 weeks of gestation, a 1-point decrease in PAPP-A was associated with an 84% increase in the likelihood of developing EOP. Following univariable analysis, multivariable logistic regression analysis identified BMI, PE history, primigravida status, MAP, free β-hCG, and PAPP-A as significant factors affecting pregnancy outcomes. Multicollinearity analysis showed acceptable VIF values (<2.5) for all variables included in the final multivariable logistic regression model. Table 3 provides a more detailed statistical analysis of these parameters.

**Table 1:** Comparison of baseline characteristics of study groups

	**Early-onset PE (*n* ═ 202)**	**Controls (*n* ═ 316)**	
**Characteristics**	**Median (IQR)/*n*(%)**	**Median (IQR)/*n*(%)**	***P* value**
Age (years)	30.0 (26.0–35.0)	31.0 (27.0–34.0)	
18–25	47 (23.3%)	50 (15.8%)	0.176
26–30	58 (28.7%)	107 (33.9%)	
31–35	60 (29.7%)	102 (32.3%)	
36–40	33 (16.3%)	47 (14.9%)	
41–45	4 (2%)	6 (1.9%)	
≥46	0 (0%)	4 (1.3%)	
BMI (kg/m^2^)	28.03 (25.07–33.26)	27.54 (25.76–29.38)	
≤18.5	0 (0.0%)	0 (0.0%)	<0.001
18.5–24.9	47 (23.3%)	49 (15.5%)	
25–29.9	77(38.1%)	198 (62.7%)	
30–34.9	38 (18.8%)	61 (19.3%)	
≥35	40 (19.8%)	8 (2.5%)	
Parity			
Nullipar	103 (51.0%)	67 (21.2%)	<0.001
Primipar	59 (29.2%)	137 (43.4%)	
Multipar	40 (19.8%)	112 (35.4%)	
DM/GDM			
(+)	34 (16.8%)	68 (21.5%)	0.191
(−)	168 (83.2%)	248 (78.5%)	
MAP (mm/Hg)	96.6 (90.0–113.3)	83.3 (73.3–86.6)	<0.001
PE/E/HT history	23 (11.4%)	4 (1.3%)	<0.001

IQR: Inter quantile range; PE: Preeclampsia; BMI: Body mass index; DM: Diabetes mellitus; GDM: Gestational diabetes mellitus; MAP: Mean arterial pressure; E: Eclampsia; HT: Hypertension.

**Table 2:** Comparison of baseline laboratory values of pregnants according to first trimester visit

	**Early-onset PE (*n* ═ 202)**	**Controls (*n* ═ 316)**	***P* value**
Hemoglobin (mg/dL)	11.70 (10.90–12.80)	12.10 (11.30–12.80)	0.054
Hematocrit (%)	35.05 (32.30–37.80)	36.05 (33.90–38.30)	0.010
Leucocytes (×10^3^/uL)	10.66 (8.40–12.60)	10.28 (8.60–12.12)	0.801
Platelet (×10^3^/uL)	215.50 (168.00–272.00)	218.00 (180.00–261.00)	0.967
Neutrophil (×10^3^/uL)	8.40 (6.54–9.93)	7.57 (6.13–9.27)	0.058
ALT (U/L)	11.1 (8.0–16.5)	10.0 (7.6–13.0)	0.002
AST (U/L)	16.0 (13.0–21.2)	15.3 (13.0–19.0)	0.218
Urea (mg/dL)	17.8 (14.7–22.0)	14.0 (12.0–18.0)	0.051
Creatinine (mg/dL)	0.56 (0.48–0.66)	0.53 (0.47–0.59)	0.001
Cholesterol (mg/dL)	198.5 (170.3–221.9)	216.0 (164.3–233.6)	0.655
LDL (mg/dL)	118.8 (103.6–145.0)	105.0 (87.3–128.1)	0.109
Triglyceride (mg/dL)	127.0 (101.6–175.8)	195.95 (148.0–229.0)	0.005
HDL (mg/dL)	55.0 (48.8–63.0)	68.1 (53.4–83.7)	<0.001
Albumin (g/dL)	3.7 (3.3–4.1)	3.8 (3.6–4.0)	0.378
Protein (g/dL)	6.6 (6.2–7.0)	6.7 (6.4–6.9)	0.731
C-reactive protein (mg/L)	0.6 (0.4–0.9)	0.7 (0.4–1.0)	0.413
Free β-hCG (mIu/mL)	21.71 (13.77–31.95)	34.06 (23.18–57.35)	<0.001
PAPP-A (MoM)	0.85 (0.59–1.11)	1.87 (1.03–2.5)	<0.001

ALT: Alanine transaminase; AST: Aspartate aminotransferase; LDL: Low-density lipoprotein; HDL: High-density lipoprotein; PE: Preeclampsia; PAPP-A: Pregnancy-associated plasma protein-A; MoM: Multiples of the median.

**Table 3:** Univariable and multivariable logistic regression analysis for the prediction of early-onset preeclampsia

	**Early-onset PE (*n* ═ 202)**						
	**Univariable analysis**	**Multivariable analysis**					
	**Odds ratio (95% CI)**	***P* value**	**Odds ratio (95% CI)**	***P* value**	**Wald**	**β-coefficiens**	VIF*
Age	0.978 (0.947–1.011)	0.184		NS			
BMI≥35 (kg/m^2^)	9.506 (4.347–20.791)	<0.001	5.842 (1.043–32.711)	0.045	7.677	1.674	NA
History of DM	0.738 (0.468–1.164)	0.192		NS			
History of PE	10.022 (3.412–29.441)	<0.001	13.900 (1.927–100.238)	0.009	19.017	2.496	NA
Primigravida	3.867 (2.629–5.687)	<0.001	2.486 (1.167–5.296)	0.018	7.264	0.998	NA
Hematocrit	0.943 (0.899–0.990)	0.018		NS			
Neutrophil	1.094 (1.026–1.167)	0.006	1.246 (1.024–1.515)	0.028	4.823	0.080	1.006
ALT	1.032 (1.016–1.049)	<0.001	1.040 (1.009–1.072)	0.012	10.255	0.060	1.015
Urea	1.126 (1.086–1.167)	<0.001		NS			
Creatinine	17.627 (3.877–80.148)	<0.001		NS			
Triglyceride	0.996 (0.995–1.001)	0.083		NS			
HDL	0.941 (0.928–0.955)	<0.001		NS			
MAP	1.324 (1.250–1.404)	<0.001	1.298 (1.205–1.398)	<0.001	68.200	0.277	1.063
Free β-hCG	0.956 (0.944–0.967)	<0.001	0.973 (0.953–0.993)	0.009	7.881	−0.027	1.010
PAPP-A	0.162 (0.113–0.232)	<0.001	0.217 (0.125–0.378)	<0.001	20.093	−0.677	1.026

*VIF values were calculated using linear regression analysis. NS: Not significant; CI: Confidence interval; BMI: Body mass index; DM: Diabetes mellitus; PE: Preeclampsia; ALT: Alaine aminotransferase; HDL: High density lipoprotein; MAP: Mean arterial pressure; PAPP-A: Pregnancy-associated plasma protein-A; VIF: Variance inflation factor.

The risk of EOP development according to PAPP-A, MAP, BMI, and DM history is demonstrated in Table 4. Prediction of EOP varied significantly within PAPP-A subgroups based on MAP levels, BMI, and DM history. When PAPP-A ≥ 0.88 and BMI < 35 kg/m^2^ were used as the reference, pregnant women with PAPP-A < 0.88 and BMI ≥ 35 kg/m^2^ had an OR of 20.410 (11.104–37.515) in the crude model and 19.945 (8.088–49.185) in the adjusted model. Using PAPP-A ≥ 0.88/MAP < 85 mmHg as a reference, pregnant women with PAPP-A < 0.88 and MAP ≥ 85 mmHg had an OR of 121.133 (54.107–272.086) in the crude model and 114.826 (51.046–258.298) in the adjusted model. Similar improvements in EOP prediction were observed in PAPP-A subgroups compared with DM history in the crude model; however, no improvements were observed in the adjusted model (Table 4).

**Table 4:** Early-onset preeclampsia (EOP) development in pregnants according to levels of PAPP-A in conjunction with MAP, BMI and DM history

	**EOP development**				
		**OR (95% CI)**		**OR (95% CI)**	
	***n*** **EOP/*n* total (%)**	**Crude model**	***P***	Adjusted model *	***P***
PAPP-A≥0.88/BMI< 35 (REF)	74/330 (22.4)	–	–	–	–
PAPP-A≥0.88/BMI≥35	14/21 (66.7)	3.098 (1.792–5.354)	<0.001	2.786 (1.173–6.616)	0.020*
PAPP-A<0.88/BMI<35	88/140 (62.9)	6.439 (3.787–10.948)	<0.001	6.541 (2.879–14.863)	<0.001*
PAPP-A<0.88/BMI≥35	26/27 (96.3)	20.410 (11.104–37.515)	<0.001	19.945 (8.088–49.185)	<0.001*
	***n*** **EOP/*n* total (%)**	**Crude model**		Adjusted model **	
		**OR(95% CI)**	***P***	**OR(95% CI)**	***P***
PAPP-A≥0.88/DM-(REF)	72/281 (25.6)	–	–	–	–
PAPP-A≥0.88/DM+	16/70 (22.9)	0.860 (0.463–1.597)	0.633	1.543 (0.655–3.632)	0.321**
PAPP-A<0.88/DM−	96/135 (71.1)	7.145 (4.517–11.302)	<0.001	5.978 (2.889–12.370)	<0.001**
PAPP-A<0.88/DM+	18/32 (56.3)	3.732 (1.766–7.885)	<0.001	2.301 (0.639–8.288)	0.202**
	***n*** **EOP/*n* total (%)**	**Crude model**		**Adjusted model**	
		**OR(95% CI)**	***P***	**OR(95% CI)**	***P***
PAPP-A≥0.88/MAP<85 mmHg (REF)	14/222 (6.3)	–	–	–	–
PAPP-A≥0.88/MAP≥85 mmHg	74/129 (57.4)	19.990 (10.499–38.058)	<0.001	19.560 (10.235–37.381)	<0.001***
PAPP-A<0.88/MAP<85 mmHg	16/57 (28.1)	5.798 (2.627–12.796)	<0.001	5.630 (2.535–12.502)	<0.001***
PAPP-A<0.88/MAP≥85 mmHg	98/110 (89.1)	121.133 (54.107–272.086)	<0.001	114.826 (51.046–258.298)	<0.001***

*Adjusted model consists of MAP and DM history; **Adjusted model consists of MAP and BMI; ***Adjusted model consists of BMI and DM history. OR: Odds ratio; CI: Confidence interval; *n*: Number; PAPP-A: Pregnancy-associated plasma protein A; DM: Diabetes mellitus; MAP: Mean arterial pressure.

We evaluated the impact of PAPP-A, MAP, and DM history on the discriminative accuracy of different prediction models (Table 5). At first, we developed a base model to recognize patients at high risk (older age, history of PE, and increased BMI) for EOP development. Pairwise analysis demonstrated that incorporating PAPP-A into the base model significantly enhanced its accuracy in predicting EOP (DBA: −0.182, *P* < 0.001). Likewise, adding MAP to the base model resulted in significantly higher accuracy in predicting EOP (DBA: −0.276, *P* < 0.001). However, no significant improvement was observed in the model’s ability to predict EOP after adding DM history (DBA: −0.011, *P* ═ 0.339) (Table 5).

**Table 5:** Impact of the PAPP-A, MAP, and DM history on the discrimination accuracy of early-onset PE development

		**AUROC curve (95% CI)**		**Pairwise analysis**					
		**Without PAPP-A**	**With PAPP-A**	**95% CI**					
**Prognostic model**				**DBA**	**SE**	**Lower**	**Upper**	**Z statistic**	***P***
Base model ═ Age, PE history and BMI≥35	EOP development	0.661 (0.610–0.712)	0.842 (0.810–0.875)	−0.182	0.206	−0.232	−0.131	−7.015	<0.001
		**AUROC curve (95% CI)**		**Pairwise Analysis**					
		**Without MAP**	**With MAP**	**95% CI**					
**Prognostic model**				**DBA**	**SE**	**Lower**	**Upper**	**Z statistic**	***P***
Base model ═ Age, PE history and BMI≥35	EOP development	0.661 (0.610–0.712)	0.937 (0.914–0.960)	−0.276	0.194	−0.328	−0.224	−10.425	<0.001
		**AUROC curve (95% CI)**		**Pairwise analysis**					
		**Without DM history**	**With DM history**	**95% CI**					
**Prognostic model**				**DBA**	**SE**	**Lower**	**Upper**	**Z statistic**	***P***
Base model ═ Age, PE history and BMI≥35	EOP development	0.661 (0.610–0.712)	0.673 (0.624–0.723)	−0.011	0.224	−0.039	0.013	−0.955	0.339

AUROC: Area under receiver operating characteristics; CI: Confidence interval; SE: Standard error; DBA: Difference between areas; PE: Preeclampsia; BMI: Body mass index; MAP: Mean arterial pressure; DM: Diabetes mellitus; PAPP-A: Pregnancy-associated plasma protein-A.

**Figure 1.**
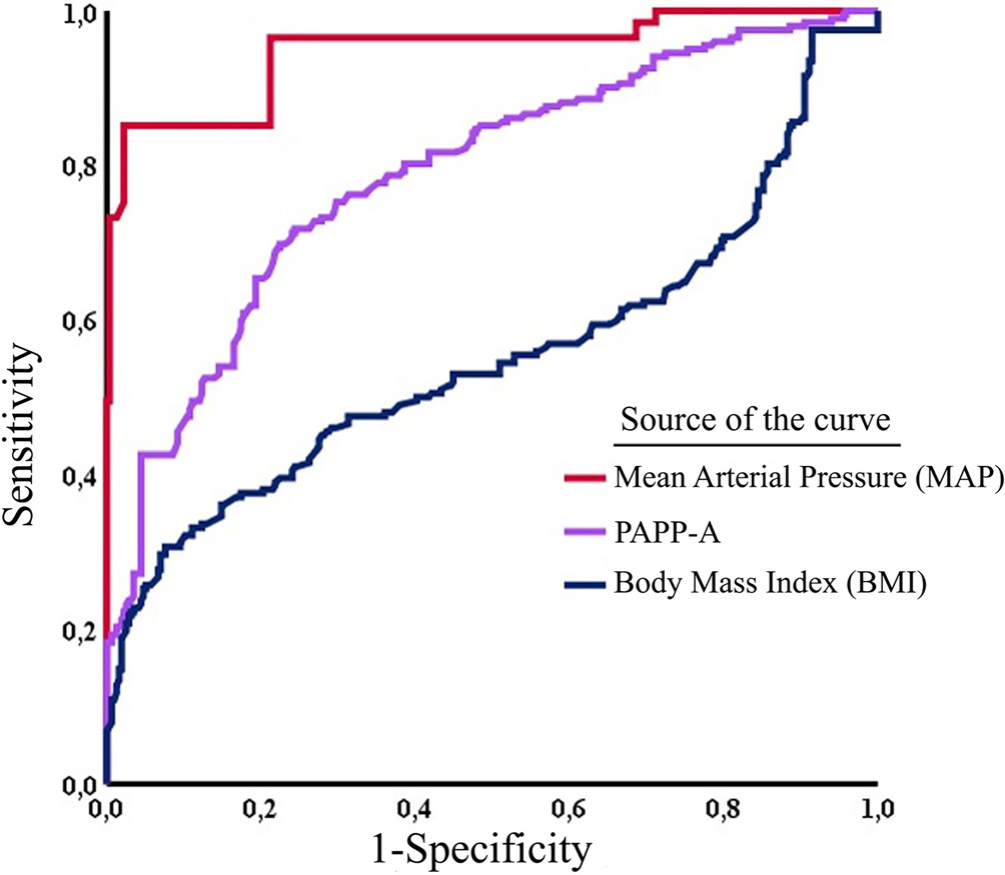
Receiver operating characteristic (ROC) curves demonstrating the predictive performance of mean arterial pressure (MAP), pregnancy-associated plasma protein-A (PAPP-A), and body mass index (BMI) for early-onset preeclampsia.

The significance of differences between AUCs was further examined through pairwise comparison in ROC curve analysis. A statistically significant difference in predicting EOP was observed between the base model + PAPP-A and the base model + MAP, with a DBA of −0.094 (*P* < 0.001) (Figure 1). Similar analyses were performed for comparisons between base model + PAPP-A vs base model + DM history (DBA: 0.169, *P* < 0.001) and base model + MAP vs base model + DM history (DBA: 0.264, *P* < 0.001) (Table 6).

**Table 6:** Pairwise comparisons of receiver operating characteristic curves

				**95% CI**			
	**AUC**	**DBA**	**SE**	**Lower**	**Upper**	**Z statistics**	***P* value**
**EOP development**							
Base model + PAPP-A vs Base model + MAP	0.842 (0.810–0.875) vs 0.937 (0.914–0.960)	−0.094	0.169	−0.132	−0.057	−4.904	<0.001
Base model + PAPP-A vs Base model + DM +	0.842 (0.810–0.875) vs 0.673 (0.624–0.723)	0.169	0.204	0.119	0.220	6.578	<0.001
Base model + MAP vs Base model + DM +	0.937 (0.914–0.960) vs 0.673 (0.624–0.723)	0.264	0.192	0.213	0.314	10.276	<0.001

AUC: Area under curve; DBA: Difference between areas; CI: Confidence interval; SE: Standard error; EOP: Early-onset preeclampsia*;* MAP: Mean arterial pressure; DM: Diabetes mellitus; PAPP-A: Pregnancy-associated plasma protein-A.

## Discussion

This study analyzed the efficiency of a novel model to examine the predictive performance of first-trimester PAPP-A and MAP in combination with several demographic and laboratory risk factors to predict EOP. As a result, we demonstrated that lower PAPP-A, higher MAP, and DM are associated with EOP development in conjunction with other demographic and clinical parameters. Classifying patients by BMI values, MAP levels, and DM existence significantly improved the first-trimester EOP prediction across the subgroups of PAPP-A in crude and adjusted logistic regression analysis. Furthermore, predictive models incorporating PAPP-A and MAP demonstrated greater accuracy compared to those that excluded these laboratory and clinical parameters. Pairwise comparisons of ROC curves demonstrated that, compared to PAPP-A (AUC: 0.842), adding MAP (AUC: 0.937) to the base model has a slight superiority in predicting EOP.

Hypertensive disorders occurring during pregnancy, particularly PE, are recognized as major causes of maternal and perinatal morbidity and mortality [11]. PE can be sub-categorized into two subclasses: EOP, which necessitates delivery before 34 weeks of gestation, and LOP, characterized by delivery at or beyond 34 weeks. Although both EOP and LOP are life-threatening conditions, the former is linked with a higher incidence of maternal and fetal complications and healthcare resource utilization [12]. Hence, early identification of pregnancies at high risk for EOP and implementing necessary measures to enhance placentation and decrease the prevalence of the disease is crucial. In this context, a great number of defined PE risk factors have been proposed by professional organizations, including the American College of Obstetricians and Gynecologists (ACOG) and the National Institute for Health and Care Excellence (NICE) [13–15]. Unfortunately, accumulating evidence suggests that PE screening using the ACOG and NICE approach exhibits diminished performance. For example, the NICE recommendation attains a detection rate of only 41% for preterm PE and 34% for term PE at a 10% false positive rate. ACOG-based recommendations also have low detection rates of 5% for preterm PE and 2% for term PE, with a false positive rate of 0.2% [15, 16].

In order to increase PE detection rates, various prediction models have been proposed in the last two decades with varying success rates or failed external validation [17–19]. Therefore, this study aimed to evaluate the single and combined performance of first-trimester maternal PAPP-A, MAP, BMI, and several clinical and biochemical prognostic factors to predict EOP. Furthermore, we also analyzed the effect of PAPP-A, MAP, and DM history on the discriminating accuracy of different models to discover an efficient prediction tool for EOP. In this context, PAPP-A is one of the most studied laboratory markers that have shown to be strongly associated with PE development. It is a syncytiotrophoblast-derived metalloproteinase that interacts with insulin-like growth factors and plays a crucial role in the invasion and growth of the placenta and the fetus [20]. In addition, recent evidence suggests that combining PAPP-A with other complementary first-trimester biomarkers may further enhance predictive performance. For instance, Xie et al. (2024) [21] demonstrated the added value of incorporating angiogenic and inflammatory serum markers in early risk stratification for PE. Low maternal PAPP-A levels have been associated with the development of pregnancy-associated adverse events, including small for gestational age infants, GDM, and PE [20, 22–24]. In line with this, we observed a decreased concentration of maternal PAPP-A at 11–13+6 weeks in patients with EOP.

Although PAPP-A is not specific to PE and its levels may also be altered in other gestational complications such as fetal growth restriction, chromosomal abnormalities, or adverse perinatal outcomes, it remains a relevant biomarker of impaired placentation and early trophoblastic dysfunction—core mechanisms in the pathogenesis of PE [25]. In our study, the predictive performance of PAPP-A significantly improved when parameters such as BMI, MAP, and the presence of DM history were combined. When the PAPP-A levels are less than 0.88 and BMI is greater than 35, EOP risk increases significantly [20.4 times (95% CI: 11.1–37.5)]. Additionally, low PAPP-A levels (<0.88) with high MAP levels substantially increased the EOP risk (adjusted OR: 114.8). In contrast, the association between low PAPP-A and DM history was not statistically significant after adjustment (adjusted OR: 2.30, *P* ═ 0.202). In this study, we also, for the first time, created a prognostic model incorporating PAPP-A and MAP with several known PE risk factors, including age, PE history, and BMI. Combining PAPP-A and MAP with a base prognostic model significantly increased the accuracy of identifying patients at high risk for EOP development. Although PAPP-A was identified as a reliable predictor of EOP in a recent study by Poon et al. [8], adding PAPP-A to the combination of maternal factors, MAP, and uterine artery measurements did not show a significant improvement in predicting LOP or gestational HT. While there are several studies in the literature depicting the role of PAPP-A, MAP, and BMI in predicting EOP, to the best of our knowledge, this is the first study to assess the effectiveness of PAPP-A combined with MAP and BMI on the discrimination accuracy of EOP in conjunction with a standardized prognostic model such as in our study. Although continuous modeling was used in multivariable analysis, dichotomization of predictors in Table 4 was intended to demonstrate combined risk strata using clinically interpretable cutoffs. We acknowledge that this approach may reduce statistical power and mask non-linear effects, and future work may explore flexible modeling strategies such as splines.

Compared to the control group, the MAPs in our EOP group were higher, which suggests the importance of arterial pressure measurements during the first trimester. Chen et al. [5] recently reported similar findings in their study in which the authors created a risk model combining MAP and PAPP-A in order to predict hypertensive disorders of pregnancy (HDP). Overall, elevated MAP and reduced PAPP-A levels during the first trimester of pregnancy have been identified as valuable markers for screening HDP. Our findings suggest that beyond traditional risk factors such as obesity and DM, first-trimester changes in maternal MAP and PAPP-A levels may reflect early subclinical cardiovascular maladaptation. This may serve as a complementary pathogenic mechanism contributing to EOP. In an elegant study by Zhang et al. [5], the predictive performance of MAP and PAPP-A was also studied. Combining maternal MAP with serum levels of PlGF and PAPP-A was found to be predictive of PE. Nevertheless, whether or not the addition of PAPP-A or MAP to traditional risk stratification tools in order to predict EOP is still controversial [5, 26–28]. This study, therefore, aims to further clarify the relationship between maternal MAP and serum levels of PAPP-A in conjunction with other traditional risk factors in predicting EOP. Discrimination accuracy of EOP was significantly enhanced after adding MAP [AUC: 0.937 (95% CI: 0.914–0.960)] to the prognostic model, which consists of older age, PE history, and BMI ≥35 kg/m^2^.

Although the integration of first-trimester MAP and PAPP-A levels into routine pregnancy screening protocols may accelerate the early identification of high-risk women and potentially enable timely preventive measures such as low-dose aspirin administration, we acknowledge several limitations of our study. First, the inclusion of additional clinical and biochemical parameters—such as uterine artery pulsatility index and serum PIGF—would have enriched the analysis, but their high cost and limited accessibility prevented their use. Second, the relatively small sample size of the EOP group compared to controls may have limited the statistical power and generalizability of our findings. Third, as this was a single-center, retrospective study, the reported AUROC and ORs may be subject to optimism bias. Internal validation via bootstrap resampling and external validation in an independent population were not performed. Likewise, calibration-in-the-large, calibration slope, and decision-curve analysis were not conducted, limiting our ability to assess model calibration and clinical utility in depth. Fourth, although the number of predictors relative to EOP cases approached the lower limit of acceptable events-per-variable (EPV), multicollinearity was formally assessed using VIF, and interpretations were made with caution. Finally, although multiple pairwise comparisons were conducted, formal correction methods such as Bonferroni or false discovery rate (FDR) adjustments were not applied, which may have increased the risk of Type I error.

## Conclusion

In conclusion, higher MAP, lower serum PAPP-A, and elevated BMI levels in conjunction with certain demographic and laboratory characteristics in the first trimester of pregnancy appear to be valuable predictors for assessing the risk of EOP. Prognostic models that combine PAPP-A, MAP, and BMI are much superior in predicting EOP compared to traditional methods based on a single or small number of variables. Combining these risk stratification tools may help to predict pregnant women at high risk for PE, enabling early diagnosis and timely interventions.
